# Supplementary material for: Viscoelastic Properties of Polyelectrolyte Multilayers Swollen with Ionic Liquid Solutions
Source: Polymers (Basel). 2019 Aug 1;11(8):1285. doi: 10.3390/polym11081285 (PMC6722675; doi:10.3390/polym11081285)
Supplement: Supplementary file 1 [file polymers-11-01285-s001.pdf]

polymers

Supplementary Materials: Viscoelastic Properties of Polyelectrolyte Multilayers Swollen with Ionic Liquid Solutions

Nagma Parveen [1,2,*], Pritam Kumar Jana [3] and Monika Schönhoff [1,*]

[1] Institute of Physical Chemistry, University of Muenster, 48149 Münster, Germany
[2] NRW Graduate School of Chemistry, University of Muenster, 48149 Münster, Germany
[3] Interdisciplinary Center for Nonlinear Phenomena and Complex Systems, Université Libre de Bruxelles, 1050 Brussels, Belgium
* Correspondence: nagma.parveen@kuleuven.be (N.P.); schoenho@uni-muenster.de (M.S.)

Kelvin-Voigt model. In the Kelvin-Voigt model a Hookean elastic spring and a Newtonian viscous damper are connected in parallel. Equation (S1) expresses the relation between the stress (σ) and the strain (ε) in a Kelvin-Voigt model. According to this model, the material deforms upon application of a constant stress and when the stress is removed the material relaxes to its original state. The elastic component (μ, elastic modulus) stores the original state of the material whereas the viscous component (η, viscosity) causes a deformation of the material from its original state, respectively.

$$\sigma = \sigma_{\mathrm{E}} + \sigma_{\mathrm{vis}} = \mu\varepsilon + \eta\frac{\mathrm{d}\varepsilon}{\mathrm{d}t} \tag{S1}$$

Viscoelasticity is often expressed with a complex dynamic modulus. To determine the dynamic modulus, an oscillatory stress is applied and the resulting strain is measured. The relation between the complex dynamic elastic modulus and complex dynamic viscosity is given in Equation (S2), where the real part of the modulus, G' corresponds to the elasticity or elastic modulus (μ) and the complex part of modulus, G'' corresponds to the viscosity (η).

$$\hat{G}(\omega) = \omega G' + i\omega G'' \tag{S2}$$

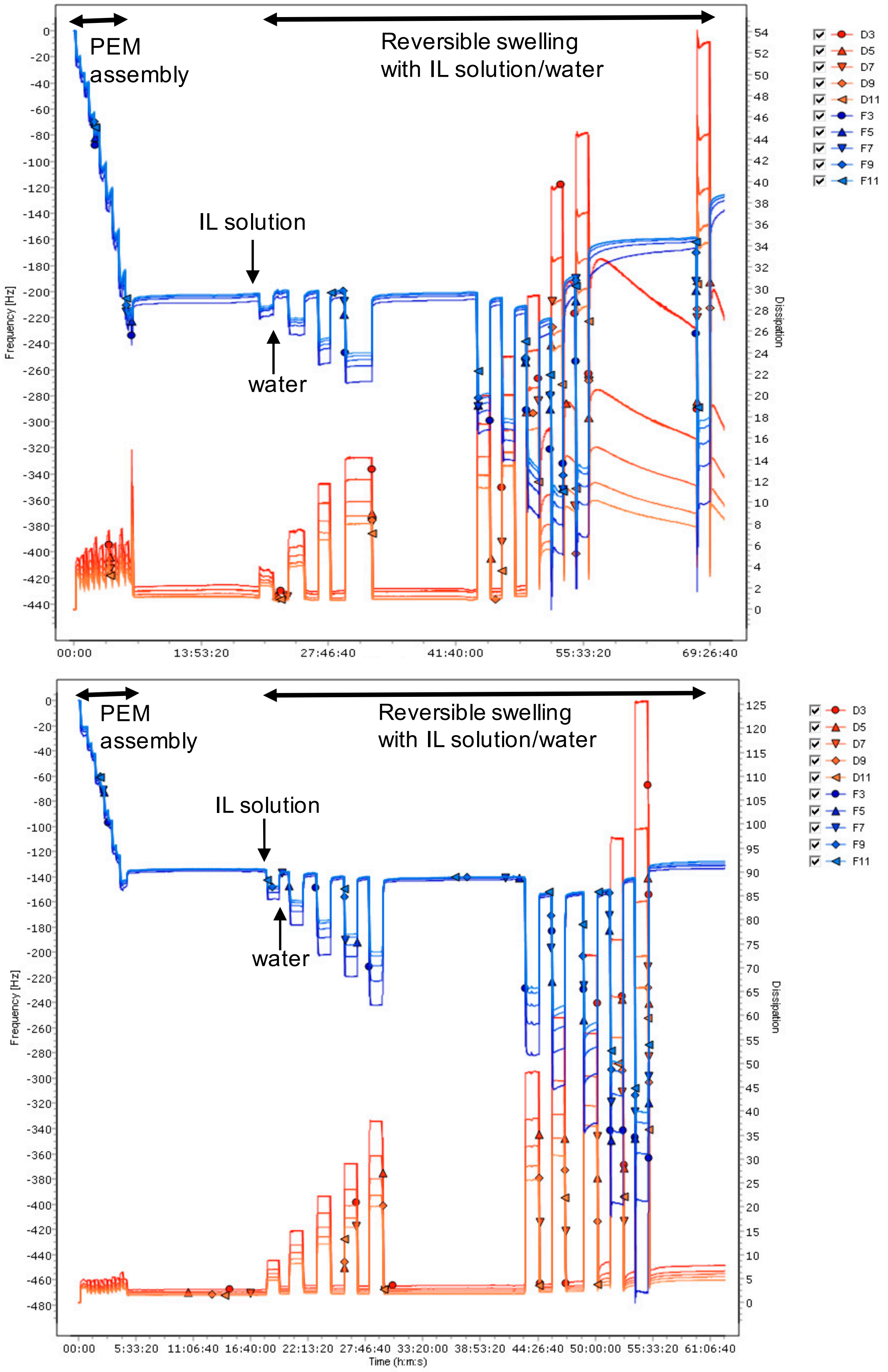

Figure S1. QCM-D shifts upon assembly and subsequent swelling of (**a**) PEI(PSS/PDADMAC)$_4$PSS and (**b**) PEI(PSS/PAH)$_4$PSS multilayers. These multilayers are composed of 10 layers of oppositely charged, i.e., PSS and either PAH or PDADMAC, and the first layer is composed of PEI which is a positively charge branched polyelectrolyte. The multilayers were exposed to aqueous solution of IL, i.e., 1-Ethyl-3methylimidazolium chloride and water subsequently. This cycle was repeated with

increasing IL concentration, i.e., 0.1 to 1.5 M and 0.3 to 3 M for PEI(PSS/PDADMAC)$_4$PSS and PEI(PSS/PAH)$_4$PSS, respectively. Here, *F* (blue lines) and *D* (red lines) stands for frequency and dissipation shifts, respectively. 3 to 11 stand for the overtone of the measured frequency and dissipation. All the measurements were performed at 22 °C.

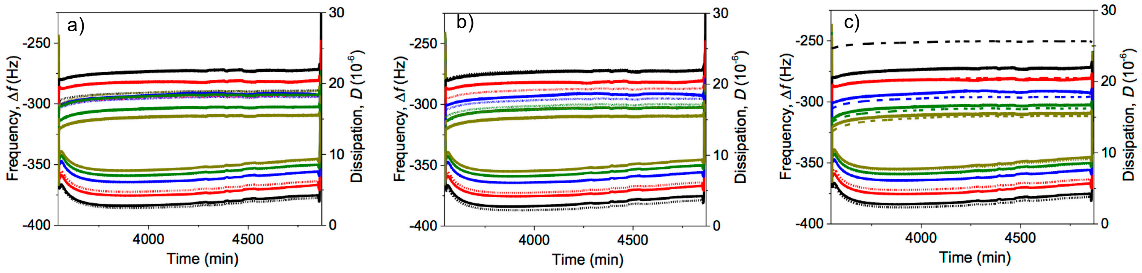

Figure S2. Comparison of the computed QCM-D shifts according to the Kelvin-Voigt modeling (dotted line) with the measured QCM-D shifts (lines) upon swelling of PEI(PSS/PDADMAC)$_4$PSS multilayer with 0.75 M EMIM solution at fluid viscosity, η_2 of (**a**) 0.0011, (**b**) 0.00125 and (**c**) 0.00135 kg m^{-1} s^{-1} while the other fixed parameters and the range of the free parameters as provided in Table 1 are the same. Bold and dashed lines are measured and modeled QCM-D responses, respectively, and the upper lines are dissipation data presented by the right axis. Black to red to blue to green lines are 3rd to 11th odd overtones of QCM-D. A best-fit of the modeled and measured shifts is obtained at η_2 of 0.00125 kg m^{-1} s^{-1}, illustrating the sensitivity of the best-fit modeling on the η_2 value.

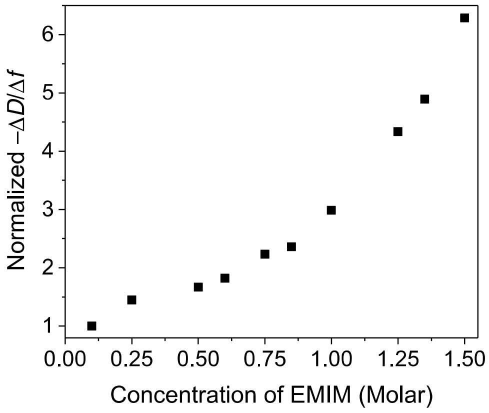

Figure S3. Normalized dissipation, ΔD to frequency, Δf ratio of a PEI(PSS/PDADMAC)$_4$PSS multilayer swollen with the given EMIM concentration. The ratio was calculated from the QCM-D shifts of 3rd overtone.
